# Changes in Nutritional Status and Sport-Specific Performance During Ramadan in Adolescent Male Volleyball Players: A Prospective Cohort Study

**DOI:** 10.3390/nu18193227

**Published:** 2026-09-30

**Authors:** Eda Elif Delice, Gülgün Ersoy

**Affiliations:** 1 Department of Nutrition and Dietetics, Institute of Health Sciences, Istanbul Medipol University, Göztepe Neighborhood, Kavacık, Atatürk Street No. 40, Beykoz, Istanbul 34810, Turkey; gersoy@medipol.edu.tr; 2 Tokat Provincial Directorate of Youth and Sports, New Neighborhood, Gaziosmanpaşa Street, No. 3, Center, Tokat 60030, Turkey

**Keywords:** adolescent, fasting adherence, Ramadan fasting, sport-specific performance, volleyball

## Abstract

**Background/Objectives**: Many adolescent athletes fast during Ramadan, similar to adults. However, research on Ramadan fasting during adolescence, a critical period for growth and development, is insufficient. This study aimed to examine changes in nutritional status and sport-specific performance during Ramadan in adolescent volleyball players and to explore whether these changes differed by Ramadan fasting adherence. **Methods**: This prospective cohort study included 35 male adolescent volleyball players (age: 16.43 ± 1.29 years, height: 1.75 ± 0.07 m, training experience: 6.20 ± 2.13 years) who fasted during Ramadan. Energy and macronutrient intake, anthropometric measurements, and sport-specific performance tests were conducted before, during, and at the end of Ramadan, and biochemical parameters were measured before and at the end of Ramadan. **Results**: Fat intake decreased during and at the end of Ramadan (1.60 ± 0.16 and 1.52 ± 0.15 g/kg BW, respectively; *p* = 0.021). Energy and macronutrient intakes were generally consistent with recommended levels, whereas fluid intake remained relatively low (1.60 ± 0.11 L, 1.35 ± 0.09 L, 1.44 ± 0.10 L, respectively). Anthropometric measurements remained unchanged. Left-hand grip strength (*p* = 0.006), 20 m sprint performance (*p* < 0.001), and agility performance (*p* = 0.002) decreased over the study period. Significant group effects were observed for 5 m, 10 m, and 20 m sprint, SJ, and standing long jump performances according to fasting adherence. Chloride, urea, and the BUN/creatinine ratio increased at the end of Ramadan, whereas albumin and potassium remained unchanged. **Conclusions**: In adolescent male volleyball players, Ramadan fasting may be associated with changes in dietary intake, certain sport-specific performance measures, and biochemical parameters potentially related to hydration status.

## 1. Introduction

During Ramadan, Muslims abstain from food and drink from dawn until sunset for 29–30 consecutive days [1]. Ramadan fasting lasts approximately 9–22 h, depending on the geographic location and the season [2,3]. Some main meals and snacks are skipped during the fasting period. Therefore, energy and nutrient intake, hydration status, body composition, and biochemical parameters may be altered during Ramadan fasting [4]. Studies of adults [5,6,7] have reported decreases in plasma LDL cholesterol, total cholesterol, and triglyceride levels; increases in HDL levels; increased fat oxidation; and decreases in body weight and body fat mass. These findings suggest that Ramadan fasting may have potential health benefits, including improvements in some metabolic health markers [1,2].

International sports competitions do not grant special exemptions for religious practices; therefore, Muslim athletes must cope with the challenges of fasting during competitions that coincide with the month of Ramadan [4,5,8]. The number of meals consumed by athletes during fasting, as well as the variety of foods, energy, fluid, and nutrient intake, varies [7,9,10,11]. Insufficient energy, fluid, and nutrient intake may alter energy substrate availability and metabolic responses, potentially contributing to fatigue, dehydration, sleep disturbances, and reductions in motivation and concentration. In such situations, sports performance is negatively affected, increasing the risk of sports-related injuries and disabilities [12,13,14,15,16,17,18].

The effects of Ramadan on nutritional status and sports performance remain unclear [4,19]. For example, in the meta-analysis of Correia et al. [20], it was found that insufficient energy, fluid, and nutrient intake among fasting athletes may lead to glycogen depletion, changes in circadian rhythms, decreased motivation, increased stress, and alterations in biochemical parameters and sports performance. Similarly, several studies have shown that fasting during Ramadan negatively affects components of sports performance, such as speed, agility, and strength [21,22,23,24,25,26,27]. However, some studies suggest that sports performance can be maintained [4,17,28,29,30] or even increased [1,31] when energy, fluid, and nutrient intake, sleep, and training intensity are balanced. Martínez-Rodríguez et al. [1] found that when adequate rest was provided in addition to an individualized hydration and nutrition plan during Ramadan, body weight and body fat were lost, while muscle mass and sports performance increased. Similarly, Güvenç [31] showed that sports performance increases when body fluid status, daily energy intake, and sleep duration are balanced, provided that regular training continues throughout Ramadan.

Many adolescent athletes, like adults, fast during Ramadan every year. Adolescence is a critical developmental period characterized by high energy and nutritional needs due to rapid growth. During Ramadan, changes in meal timing and eating patterns may challenge the ability of adolescent athletes to meet their daily energy, fluid, and nutrient requirements. Therefore, ensuring adequate energy, nutrient, and fluid intake is particularly important for adolescent athletes during Ramadan. However, research on adolescent athletes during Ramadan remains limited, particularly regarding whether temporal changes in nutritional status and sport-specific performance differ according to fasting adherence [31,32,33]. This study aimed to examine changes in nutritional status and sport-specific performance during Ramadan in adolescent volleyball players and to explore whether these changes differed according to Ramadan fasting adherence. It was hypothesized that nutritional status and sport-specific performance would change during Ramadan and that these changes would differ according to fasting adherence.

## 2. Materials and Methods

### 2.1. Participants

G*Power 3.1 was used to determine the sample size; the minimum required was 30 participants. This prospective cohort study included 35 male adolescent volleyball players (age: 16.43 ± 1.29 years, height: 1.75 ± 0.07 m, training experience: 6.20 ± 2.13 years) who voluntarily observed Ramadan fasting during the study period. The participants fulfilled the following criteria: (i) having observed Ramadan fasting for at least the previous two years; (ii) exercising at least 3 days a week (≈90 min) and maintaining a consistent workout intensity; (iii) not having a chronic disease; and (iv) not taking medication regularly. Supplement use was assessed during the study, and participants reported not using nutritional supplements. Before starting the study, a written informed consent form outlining the purpose, scope, steps, potential benefits, confidentiality, and voluntary participation was distributed to the volleyball players. Informed consent was obtained from all subjects involved in the study. Signed informed consent was obtained from the parents of athletes under 18 years of age, and from the athletes themselves if they were 18 or older. Approval was obtained from the Istanbul Medipol University Non-Interventional Research Ethics Committee (Decision No: 896, 2 September 2021) before the start of the study. All methods were performed in accordance with the relevant guidelines and regulations and the Declaration of Helsinki of 1975.

### 2.2. Study Design

**Figure 1:**
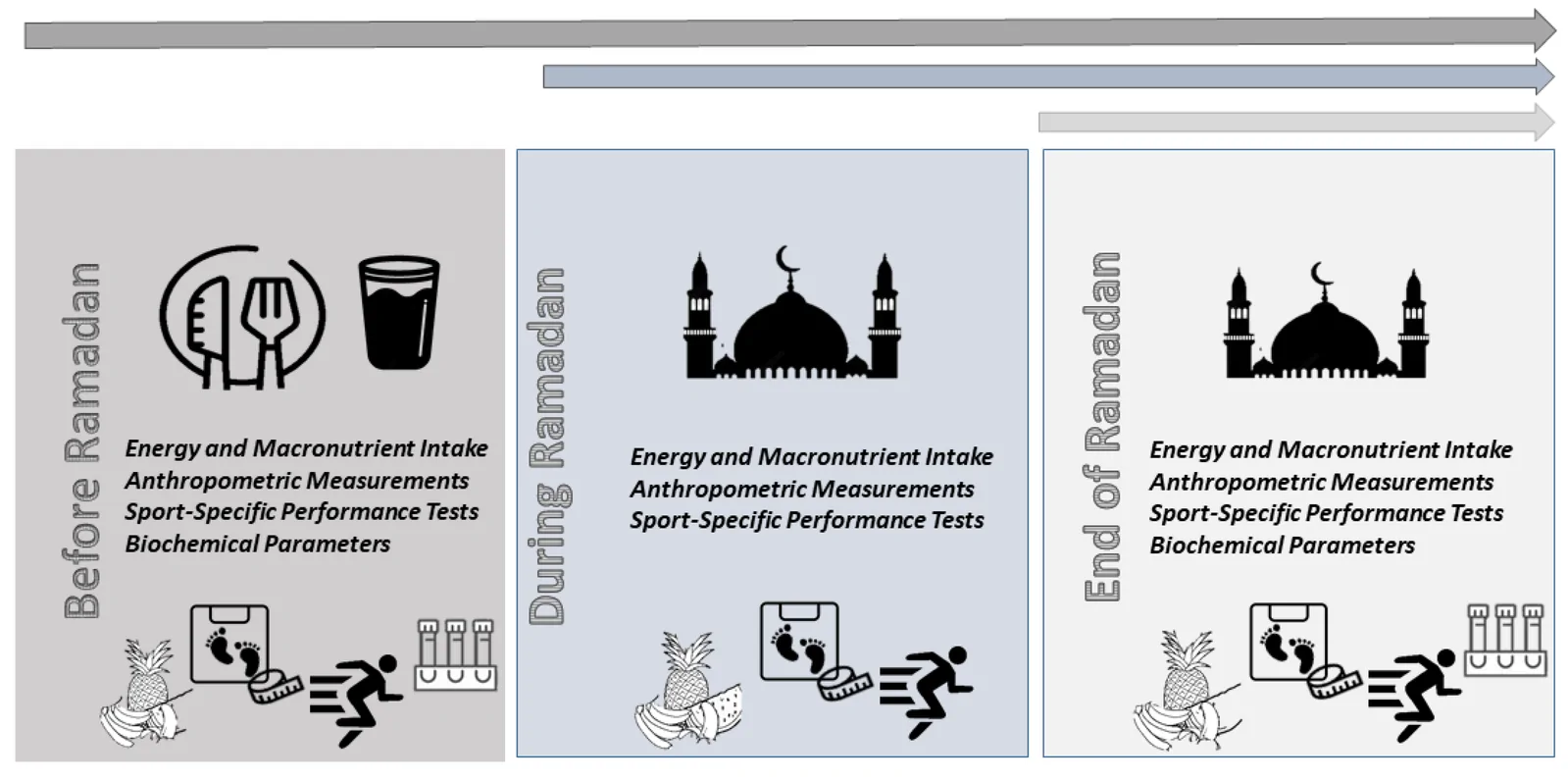
Study Design and Periods.

The research was conducted in Turkey between April and May 2022. Energy and macronutrient intake, anthropometric measurements, and sport-specific performance tests were examined in three periods: before, during, and at the end of Ramadan. Additionally, blood samples were collected at two time points: before and at the end of Ramadan (Figure 1). Each assessment period was completed within three consecutive days. Measurements before Ramadan were performed three days before the beginning of Ramadan; measurements during Ramadan were taken on the 14th, 15th, and 16th days; and measurements at the end of Ramadan were taken on the 28th, 29th, and 30th days.

The volleyball players continued their routine training sessions after Iftar (the evening meal that breaks the daily Ramadan fast) throughout Ramadan and refrained from training for 24 h before all measurements. Training schedules were determined by the coaching staff and were not modified for the study. All measurements were performed at the same time intervals and in the same order to minimize the effects of circadian variation. On the evening (between 9:00 and 10:00 PM) of the first day of each measurement period, the volleyball players arrived at the indoor sports hall, and anthropometric measurements were taken first. Subsequently, sport-specific performance tests were administered under the supervision of an expert faculty member from the Department of Physical Education and Sports at the Faculty of Sports Sciences, Gaziosmanpaşa University, Tokat. The athletes performed sprint and agility tests after a standard 15 min warm-up. To avoid negatively impacting performance, after a rest day, jumping and strength tests were conducted the following day.

Blood samples were collected from volleyball players in two periods, before and at the end of Ramadan, at the Tokat Gaziosmanpaşa University Research and Application Hospital, Faculty of Medicine. Blood samples were collected on the first day of each period (between 6:00 and 7:00 AM), after at least 8 h of fasting. Participants were instructed not to consume any food or beverages other than water after 10:00 PM on the previous evening. The collected data were analyzed for urea, creatinine, albumin, potassium, and chloride, which were selected as biochemical indicators potentially related to hydration status and fluid balance.

### 2.3. Procedures

#### 2.3.1. Energy and Macronutrient Intake

The dietary questionnaire used in this study was specifically developed by the research team for the purposes of this study. Before the start of the study, participants were provided with the dietary questionnaire and instructed to complete a prospective dietary record of their food and fluid intake using standard household measures and portion descriptions (e.g., glass, cup, tablespoon, teaspoon, slice, and portion) based on the Turkish Nutrition Guide (TÜBER) [34]. Food and fluid intake records were collected for 9 days, with 3 days of recording (including 1 weekend day) in each study period: before, during, and at the end of Ramadan. The researchers conducted regular telephone interviews with participants to improve the accuracy of the records and clarify incomplete or ambiguous food descriptions and portion sizes. The data were entered into the Nutrition Information System (BeBIS 9, Turkish Version; Ebispro for Windows, Stuttgart, Germany). The BeBIS 9 software converted the reported amounts into gram weights based on the corresponding standard portion sizes in its database, and the average daily energy and nutrient intakes were analyzed. Food and fluid intake were compared to American College of Sports Medicine (ACSM) recommendations [35] and adolescent-specific guidelines [36].

#### 2.3.2. Anthropometric Measurements

Body weight (kg) was measured using a calibrated digital scale (TANITA SC-330, Tokyo, Japan); height (m) was measured using a stadiometer (MESİLİFE, SW-G06B, Istanbul, Turkey), and neck circumference (cm), waist circumference (cm), and hip circumference (cm) were measured using a non-flexible tape measure with 1 mm intervals. All measurements were performed at the same time of day, with no vigorous exercise within 24 h before measurement and after bladder emptying. All anthropometric measurements were performed by the same researcher. Waist-to-hip ratio (WHR) was calculated from the anthropometric measurements.

#### 2.3.3. Sport-Specific Performance Tests

Strength was assessed using the handgrip strength test, speed using the 5, 10, and 20 m sprint tests, agility using the Agility T-Test, and lower-body power using the Squat Jump (SJ) and standing long jump tests. All performance tests were standardized and performed according to previously validated protocols, and participants completed one familiarization session for all performance tests before baseline testing. Testing was conducted at the same time of day (3–4 h after iftar). Two trials were performed for each test, with at least a 5 min rest interval between trials, and the best performance was recorded. Participants wore the same running shoes and clothing throughout all testing sessions and received standardized verbal encouragement during each test.

##### Hand Grip Strength Test

Volleyball players performed the hand grip strength test according to the protocol described by Mhenni et al. [37]. The participants stood with their arms at their sides and squeezed a digital calibrated hand dynamometer (Baseline Digital Hand Dynamometer 12-0286, Hong Kong) as hard as they could. Separate measurements were taken for the right and left arms.

##### Sprint Tests (5 m, 10 m, 20 m)

Volleyball players performed sprint tests according to the protocol described by Bouzid et al. [38]. Photocells (Microgate Witty, Bolzano, Italy) were placed at the start and finish points of the 5 m, 10 m, and 20 m distances. After the test protocol was explained to the participants, they were told to run as fast as possible at distances of 5 m, 10 m, and 20 m. Volleyball players started running 1 m behind the starting photocell upon a verbal signal. The time between the photocells was measured.

##### Agility T-Test

Volleyball players performed the Agility T-test according to the protocol described by Guembri et al. [39]. The participants began the test by standing sideways at the starting point of two cones placed 4.57 m apart on either side of the photocell gate (Microgate Witty, Bolzano, Italy). They first ran to the cone on the right and touched it with their right hand, then went to the cone on the left and touched it with their left hand, returned to the starting point, and completed the test. The time from the beginning to the end of the application was determined as the test completion time.

##### Squat Jump (SJ) Test

Volleyball players performed the Squat Jump (SJ) Test according to the protocol described by Aloui et al. [40]. The participants stood on a jump mat (Microgate Witty Jump Mat, Bolzano, Italy) with their feet shoulder-width apart, assumed a semi-squat, and jumped as high as possible. The digital mat measured and recorded the highest jump achieved.

##### Standing Long Jump Test

Volleyball players performed the standing long jump test according to the protocol described by Guembri et al. [39]. The participants performed the jump from a semi-squat position, aiming to reach the farthest possible distance without stepping on the starting line. The distance from the starting point to the point where the heel touches the ground after jumping was measured. If they lost their balance while landing and fell forward or backward, or if they used their hands to hold on to the ground, their measurements were considered invalid.

#### 2.3.4. Biochemical Parameters

Blood samples were collected from a forearm vein 5 min after participants had rested in a seated position. Venous blood samples of approximately 5 mL were collected in gel tubes. After approximately 30 min of clotting, the blood was centrifuged at 3500 rpm for 10 min at room temperature (20–25 °C) using a refrigerated centrifuge (ROTANTA 460 R, Hettich, Tuttlingen, Germany). After centrifugation, an automated analyzer (Roche cobas c501, Rotkreuz, Switzerland) measured the concentrations of the biochemical parameters. Urea (BUN) concentrations were determined with a kinetic enzymatic method, while albumin concentrations were determined using a colorimetric method. Creatinine concentrations were determined using the Jaffé method. Potassium and chloride concentrations were determined by potentiometry. In addition, the BUN/creatinine ratio was calculated based on the measured urea and creatinine concentrations.

### 2.4. Statistical Analysis

Statistical analyses were conducted using IBM SPSS Statistics for Windows, Version 27.0 (IBM Corp., Armonk, NY, USA). Normality was evaluated using Shapiro–Wilk tests together with visual inspection of Q–Q plots. Variables that did not demonstrate normal distribution (e.g., energy intake, 20 m sprint time, urea, and BUN/creatinine ratio) were log10-transformed before inferential analyses. Among competing covariance structures, the unstructured covariance matrix demonstrated the best model fit according to Akaike’s Information Criterion (AIC). Considering the unequal group sizes (*n* = 13, maintained fasting throughout Ramadan; *n* = 15, interrupted fasting for 1 to 7 days; and *n* = 7, interrupted fasting for 8 to 16 days) and the flexibility of mixed models for repeated-measures data (before, during, and at the end of Ramadan), Linear Mixed Models (LMMs) were employed to examine the effects of time, group, and the Time × Group interaction. In all LMM analyses, Restricted Maximum Likelihood (REML) estimation was used, and denominator degrees of freedom were calculated using the Satterthwaite approximation. Time, group, and the Time × Group interaction were included as fixed effects, whereas participant-specific intercepts were treated as random effects to account for inter-individual variability. Given the relatively small sample size, random intercept models were preferred to avoid model overparameterization. For blood parameters, time was modeled with two levels only (before and at the end of Ramadan). When significant main effects or Time × Group interaction effects were detected, Bonferroni-adjusted pairwise comparisons were performed. Effect sizes for the fixed effects were reported as semi-partial R^2^ (R^2^β), calculated using the approach proposed by Edwards et al. [41]. Results are presented as estimated marginal means (EMM) ± standard error (SE) with 95% confidence intervals (95% CI). Statistical significance was set at *p* < 0.05.

## 3. Results

Table 1 presents the estimated marginal means for energy, macronutrient, and fluid intake across the study period. Significant main effects of time were found for fat (g/kg BW) [F = 4.34, *p* = 0.021, R^2^β ≤ 0.001] and fluid (L) [F = 3.44, *p* = 0.044, R^2^β = 0.009] intake. Estimated marginal means showed decreased fat and fluid intake during and at the end of Ramadan compared with the period before Ramadan. No significant main effect of time was found for energy (kcal) [F = 2.99, *p* = 0.064, R^2^β = 0.002], carbohydrate (g/kg BW) [F = 2.46, *p* = 0.101, R^2^β = 0.007], and protein (g/kg BW) [F = 3.01, *p* = 0.063, R^2^β ≤ 0.001] intake. Neither the main effect of group nor the Time × Group interaction reached statistical significance for any nutritional variable (all *p* > 0.05). Furthermore, energy, carbohydrate, protein, and fat intakes were generally consistent with recommended levels for adolescent athletes, whereas fluid intake remained below recommended levels throughout the study period.

Table 2 presents the estimated marginal means for anthropometric measurements across the study period. No significant main effect of time was observed for body weight (kg) [F = 0.94, *p* = 0.399, R^2^β ≤ 0.001], neck circumference (cm) [F = 1.95, *p* = 0.159, R^2^β = 0.005], or WHR [F = 0.69, *p* = 0.509, R^2^β = 0.005]. Neither the main effect of group nor the Time × Group interaction reached statistical significance for any anthropometric measurement (all *p* > 0.05).

**Figure 2:**
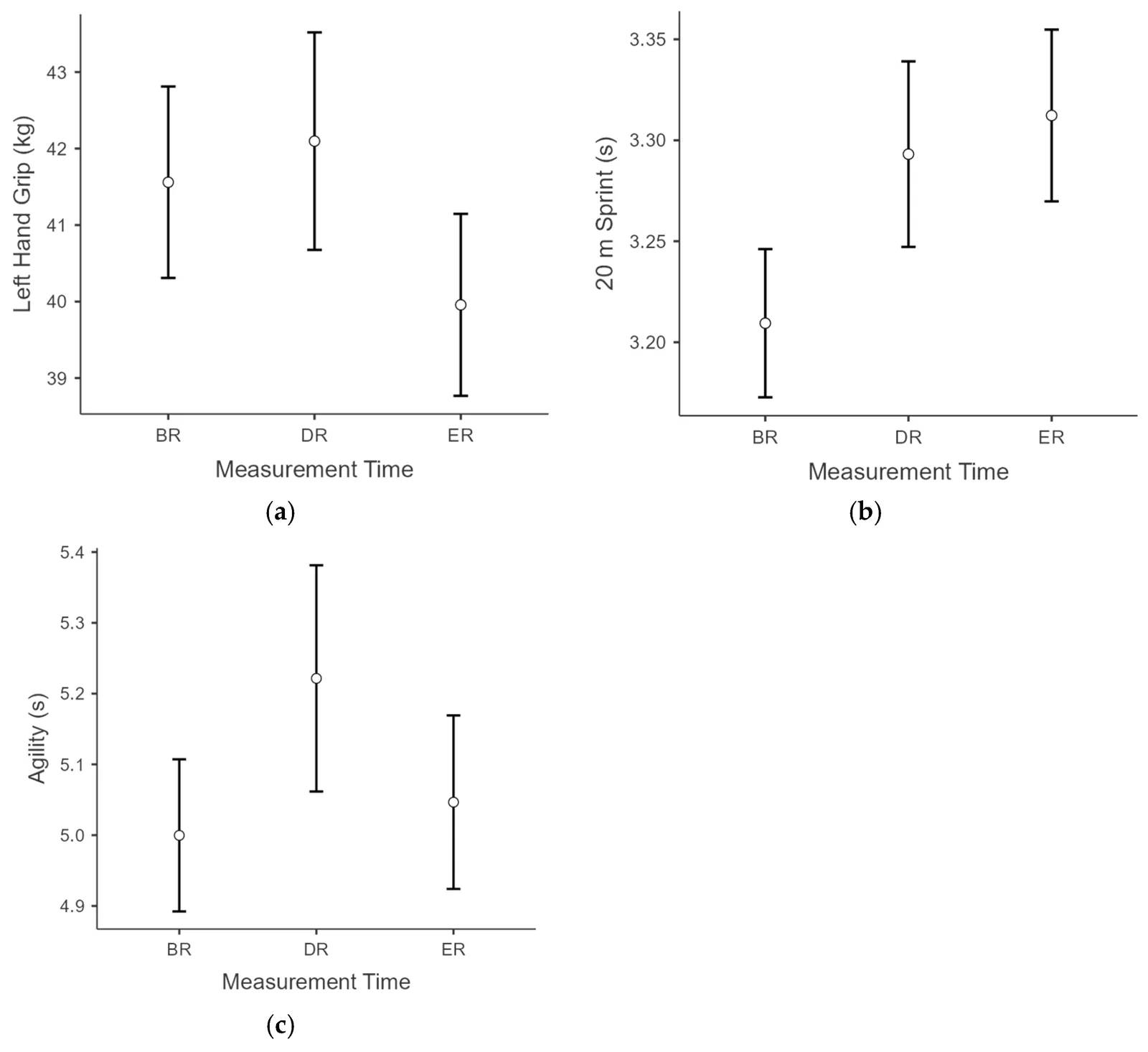
Changes in sport-specific performance measures before, during, and at the end of Ramadan: (**a**) Left-hand grip strength; (**b**) 20 m sprint time; and (**c**) Agility time. Data are presented as EMMs ± SE. BR = Before Ramadan; DR = During Ramadan; ER = End of Ramadan.

Table 3 presents the estimated marginal means for sport-specific performance measures across the study period. Significant main effects of time were observed for left-hand grip strength [F = 5.96, *p* = 0.006, R^2^β = 0.002], 20 m sprint [F = 30.78, *p* < 0.001, R^2^β = 0.022], and agility [F = 7.89, *p* = 0.002, R^2^β = 0.064]. Estimated marginal means indicated that left-hand grip strength was lower at the end of Ramadan than before and during Ramadan, whereas 20 m sprint time progressively increased across the study period. In contrast, agility time increased during Ramadan but approached baseline levels by the end of Ramadan (Figure 2). Significant main effects of group were observed for the 5 m sprint [F = 3.59, *p* = 0.039, R^2^β = 0.028], 10 m sprint [F = 3.37, *p* = 0.047, R^2^β = 0.046], 20 m sprint [F = 3.65, *p* = 0.037, R^2^β = 0.052], SJ [F = 3.86, *p* = 0.032, R^2^β = 0.063], and standing long jump [F = 4.37, *p* = 0.021, R^2^β = 0.042], indicating that performance differed according to fasting adherence group. No significant Time × Group interactions were observed for any sport-specific performance measure (all *p* > 0.05).

Table 4 presents the estimated marginal means for biochemical parameters before and at the end of Ramadan. Significant main effects of time were observed for chloride [F = 5.34, *p* = 0.027, R^2^β = 0.011], urea (BUN) [F = 12.65, *p* = 0.001, R^2^β = 0.069], and the BUN/creatinine ratio [F = 18.77, *p* < 0.001, R^2^β = 0.099]. Estimated marginal means indicated that chloride, urea (BUN), and the BUN/creatinine ratio increased at the end of Ramadan compared with before Ramadan. No significant main effect of time was observed for albumin [F = 0.23, *p* = 0.635, R^2^β = 0.007] or potassium [F = 0.44, *p* = 0.513, R^2^β = 0.005]. Neither the main effect of group nor the Time × Group interaction reached statistical significance for any biochemical parameter (all *p* > 0.05).

## 4. Discussion

In the present study, fat and fluid intake decreased during and at the end of Ramadan, whereas no significant changes were observed in energy, carbohydrate, or protein intakes. Energy and macronutrient intakes were generally consistent with recommended levels during Ramadan fasting, whereas fluid intake remained relatively low throughout the study period. Anthropometric measurements remained unchanged throughout the study period. Left-hand grip strength and 20 m sprint performance decreased at the end of Ramadan. Agility performance declined during Ramadan but approached baseline levels by the end of Ramadan. Chloride, urea (BUN), and the BUN/creatinine ratio increased at the end of Ramadan, whereas albumin and potassium remained unchanged. Overall, these findings suggest that Ramadan fasting may be associated with changes in dietary intake, sport-specific performance measures, and biochemical markers potentially related to hydration status, while anthropometric measurements remained unchanged throughout the study period. In light of these findings, it can be stated that particular attention should be paid to ensuring adequate energy, macronutrient, and fluid intake during Ramadan to support nutritional needs and training performance, especially in adolescent athletes. Furthermore, since growth and maturation processes can influence nutritional requirements and physiological responses, the fact that the participants were adolescents should be taken into account when evaluating these findings, and it should be borne in mind that the results may differ from those observed in adult athletes.

During Ramadan fasting, energy, carbohydrate, protein, and fat intakes among athletes may change depending on the number of meals consumed daily and changes in eating habits [1]. While daily energy intake (kcal) remained unchanged in studies by Khemila et al. [13], Güvenç [31], and Aloui et al. [40], it decreased at the end of Ramadan in studies by Chennaoui et al. [23] and Hammouda et al. [42,43]. In a study by Bouhlel et al. [44], carbohydrate (g/day) and protein (g/day) intakes, in addition to daily energy intake (kcal), also decreased by the end of Ramadan. In the study by Bouzid et al. [38], energy (kcal/day), carbohydrate (g/day), protein (g/day), and fat (g/day) intakes did not change. Similarly, in the study by Chaouachi et al. [45], energy (kcal/day), carbohydrate (g/kg BW), protein (g/kg BW), and fat (g/kg BW) intakes did not change. In contrast, Trabelsi et al. [46] found that energy (kcal/day), protein (g/kg BW), and fat (g/kg BW) intakes did not change at the end of Ramadan, whereas carbohydrate intake (g/kg BW) increased. In this study, energy (kcal), carbohydrate (g/kg BW), protein (g/kg BW), and fat (g/kg BW) intakes were generally consistent with the recommended levels for adolescent athletes during Ramadan fasting. While fat intake decreased significantly during and at the end of Ramadan, no significant changes were observed in energy, carbohydrate, or protein intakes (Table 1). The reduction in fat intake may reflect changes in food choices and meal patterns during Ramadan, including a possible reduction in the consumption of fat-rich foods.

Changes in energy and nutrient intake during Ramadan fasting may affect anthropometric measurements in athletes [4,5,9]. In the study by Guembri et al. [47], body weight increased in athletes at the end of Ramadan, whereas in the studies by Trabelsi et al. [48,49], it decreased. Similarly, waist and hip circumference decreased in the study by Al-Jafar et al. [50], while WHR decreased in the study by Attarzadeh Hosseini et al. [51]. In contrast, anthropometric measurements did not change in the studies by Khemila et al. [13], Meckel et al. [21], Chennaoui et al. [23], Karli et al. [29], Güvenç [31], and Zarrouk et al. [52]. In this study, no significant changes were observed in body weight, neck circumference, or WHR before, during, and at the end of Ramadan (Table 2). The absence of a significant change in total energy intake may have contributed to the maintenance of anthropometric measurements during Ramadan, despite the observed decrease in fat intake.

Ramadan fasting may have an impact on sports performance components such as sprint, agility, jumping and strength in athletes [8]. In the studies by Zerguini et al. [26], Mhenni et al. [37], Bouzid et al. [38] and Aloui et al. [40], strength, jumping and sprint performances were negatively affected at the end of Ramadan. In contrast, a study by Rebaï et al. [53], demonstrated that a recovery period characterized by reduced training volume during Ramadan could lead to improvements in muscle strength and jump performance. On the other hand, jumping, sprint, and strength performances did not change in the studies of Chaouachi et al. [30], Guembri et al. [47]. In this study, no significant temporal differences were observed in right-hand grip strength; however, left-hand grip strength was lower at the end of Ramadan than during and before Ramadan (Table 3). This differential response between the two hands may be related to changes in physical activity and fluid intake during Ramadan, which may have influenced the strength capacity of the less frequently used hand.

In this study, no significant temporal changes were observed in 5 m or 10 m sprint performance. In contrast, 20 m sprint performance, which was at its best before Ramadan, gradually decreased until the end of Ramadan (Table 3). The decline in 20 m sprint performance may have been partly related to relatively low fluid intake and possible changes in physical activity during Ramadan fasting, while the absence of significant changes in 5 m and 10 m sprint performance may indicate that performance over longer sprint distances could be more sensitive to the physiological changes associated with Ramadan fasting. Significant group effects were observed for all sprint performance measures, indicating that sprint performance differed according to fasting adherence group. Based on the present data, it is plausible that greater adherence to Ramadan fasting could be associated with greater declines in sprint performance, whereas lower adherence could be associated with better maintenance of performance; however, this hypothesis requires further investigation in future studies.

In this study, it was observed that the lowest Agility performance was recorded during Ramadan, followed by a partial recovery at the end of Ramadan (Table 3). Agility performance, which requires rapid changes in direction and can be affected by physiological and cognitive factors, may have been influenced by changes in hydration status and circadian rhythms during Ramadan fasting. The partial improvement seen at the end of Ramadan may suggest a return toward baseline performance and possible adaptation to fasting.

In this study, no significant temporal changes were observed in the SJ and standing long jump tests before, during, and at the end of Ramadan. However, significant group effects were observed for both jumping tests, indicating that jumping performance differed according to the fasting adherence group (Table 3). Based on the present data, it is plausible that greater adherence to Ramadan fasting could be associated with greater declines in jumping performance, whereas lower adherence could be associated with better maintenance of performance; however, these hypotheses require further investigation in future studies.

Ramadan fasting may lead to changes in biochemical parameters in athletes, particularly those related to hydration status, due to prolonged periods of fluid restriction and changes in meal patterns [7,9]. In the studies by Trabelsi et al. [46,48], total water intake (L) decreased at the end of Ramadan, while plasma urea, creatinine, and chloride concentrations increased. In contrast, Bouhlel et al. [44] reported that plasma urea levels decreased at the end of Ramadan despite a decrease in fluid intake, whereas creatinine levels remained unchanged. In the study by Chaouachi et al. [45], plasma albumin levels remained unchanged at the end of Ramadan. In this study, chloride, urea (BUN), and the BUN/creatinine ratio increased at the end of Ramadan compared with before Ramadan (Table 4). The observed increases in these parameters may suggest possible changes in hydration status during Ramadan fasting. The inability to consume fluids during the day while fasting in Ramadan may have limited daily fluid intake and may have contributed to the observed changes in these biochemical parameters. Indeed, in this study, fluid intake, which was already insufficient before Ramadan, decreased further during Ramadan fasting and remained below recommended levels throughout the study period. However, the absence of significant changes in albumin and potassium levels may be consistent with possible mild changes in hydration status rather than severe dehydration.

This study has several limitations. First, a non-fasting control group was not included. The study aimed to investigate temporal changes according to fasting adherence under real-world conditions; therefore, participants with different levels of fasting adherence were included in the cohort from the beginning and in the statistical analyses. The findings should not be interpreted as a comparison between fasting and non-fasting athletes. Second, female athletes were excluded from the study due to considerations related to menstrual cycles and the use of hormonal medications. Therefore, the results cannot be generalized to female adolescent athletes. Third, the sample size was relatively small, which may limit the statistical power and generalizability of the findings. Future studies with larger and more diverse samples are warranted to confirm these findings and provide a more comprehensive understanding of the effects of Ramadan fasting on adolescent athletes. Finally, the physical performance tests assessed short-duration, high-intensity performance but did not evaluate repeated high-intensity efforts or endurance demands during volleyball play. In addition, performance testing was conducted 3–4 h after iftar; therefore, it may not fully reflect performance during the fasting period. Future studies should incorporate repeated-sprint, aerobic capacity, or sport-specific intermittent exercise protocols and assess performance at different time points relative to fasting and iftar to better reflect the physiological demands of volleyball.

## 5. Conclusions

In adolescent male volleyball players, energy and macronutrient intake remained adequate during Ramadan fasting, whereas fluid intake remained relatively low throughout the study period. These findings suggest that Ramadan fasting may be associated with changes in dietary intake, sport-specific performance measures, and biochemical parameters potentially related to hydration status, while anthropometric measurements remained unchanged throughout the study period.

## Figures and Tables

**Table 1:** EMMs for energy, macronutrient, and fluid intake before, during, and at the end of Ramadan.

Variable	Before Ramadan EMM ± SE [95% CI]	During Ramadan EMM ± SE [95% CI]	End of Ramadan EMM ± SE [95% CI]	Time *F*, *p*, *R*^2^*β*	Group *F*, *p*, *R*^2^*β*	Time × Group *F*, *p*, *R*^2^*β*
Energy (kcal)	3046.09 ± 189.91 [2659.26, 3432.91]	2717.47 ± 188.54 [2333.44, 3101.51]	2702.63 ± 164.22 [2368.13, 3037.13]	2.99, 0.064, 0.002	0.11, 0.895, 0.009	0.87, 0.492, 0.019
Carbohydrate (g/kg BW)	5.60 ± 0.52 [4.68, 6.70]	5.02 ± 0.46 [4.20, 6.00]	5.32 ± 0.50 [4.44, 6.37]	2.46, 0.101, 0.007	0.27, 0.765, 0.003	0.93, 0.459, 0.008
Protein (g/kg BW)	1.55 ± 0.13 [1.32, 1.82]	1.34 ± 0.11 [1.14, 1.58]	1.34 ± 0.11 [1.14, 1.57]	3.01, 0.063, <0.001	0.01, 0.990, 0.007	1.11, 0.369, 0.015
Fat (g/kg BW)	1.88 ± 0.19 [1.55, 2.29]	1.60 ± 0.16 [1.32, 1.94]	1.52 ± 0.15 [1.25, 1.84]	4.34, **0.021**, <0.001	0.35, 0.707, 0.021	1.27, 0.302, 0.015
Fluid Intake (L)	1.60 ± 0.11 [1.40, 1.83]	1.35 ± 0.09 [1.18, 1.54]	1.44 ± 0.10 [1.26, 1.65]	3.44, **0.044**, 0.009	0.16, 0.853, 0.014	2.61, 0.054, 0.047

Note. BW = Body Weight; EMM = Estimated Marginal Mean; SE = Standard Error; CI = Confidence Interval; R^2^β = Semi-partial R^2^ for fixed effects. F-statistics and *p*-values were obtained from linear mixed models (LMMs). Bold *p*-values indicate statistical significance (*p* < 0.05).

**Table 2:** EMMs for anthropometric measurements before, during, and at the end of Ramadan.

Variable	Before Ramadan EMM ± SE [95% CI]	During Ramadan EMM ± SE [95% CI]	End of Ramadan EMM ± SE [95% CI]	Time *F*, *p*, *R*^2^*β*	Group *F*, *p*, *R*^2^*β*	Time × Group *F*, *p*, *R*^2^*β*
Body Weight (kg)	62.74 ± 2.20 [58.26, 67.22]	62.83 ± 2.24 [58.27, 67.40]	62.63 ± 2.18 [58.20, 67.06]	0.94, 0.399, <0.001	0.53, 0.592, 0.010	0.80, 0.534, <0.001
Neck Circumference (cm)	34.80 ± 0.34 [34.12, 35.48]	35.09 ± 0.37 [34.33, 35.85]	35.25 ± 0.34 [34.56, 35.94]	1.95, 0.159, 0.005	0.38, 0.685, 0.005	0.26, 0.901, 0.002
Waist-to-Hip Ratio (WHR)	0.81 ± 0.01 [0.79, 0.82]	0.81 ± 0.01 [0.80, 0.83]	0.81 ± 0.01 [0.80, 0.82]	0.69, 0.509, 0.005	0.41, 0.668, 0.006	0.18, 0.947, 0.002

Note. EMM = Estimated Marginal Mean; SE = Standard Error; CI = Confidence Interval; R^2^β = Semi-partial R^2^ for fixed effects. F-statistics and *p*-values were obtained from linear mixed models (LMMs).

**Table 3:** EMMs for sport-specific performance measures before, during, and at the end of Ramadan.

Variable	Before Ramadan EMM ± SE [95% CI]	During Ramadan EMM ± SE [95% CI]	End of Ramadan EMM ± SE [95% CI]	Time *F*, *p*, *R*^2^*β*	Group *F*, *p*, *R*^2^*β*	Time × Group *F*, *p*, *R*^2^*β*
Right-Hand Grip (kg)	44.61 ± 1.17 [42.23, 46.99]	45.04 ± 1.41 [42.17, 47.92]	43.96 ± 1.41 [41.08, 46.83]	1.31, 0.285, 0.002	1.75, 0.190, 0.031	1.16, 0.347, 0.005
Left-Hand Grip (kg)	42.47 ± 1.25 [39.93, 45.02]	43.02 ± 1.43 [40.12, 45.92]	40.66 ± 1.22 [38.18, 43.15]	5.96, **0.006**, 0.002	2.90, 0.070, 0.051	0.60, 0.663, 0.004
5 m Sprint (s)	1.01 ± 0.02 [0.97, 1.05]	1.02 ± 0.02 [0.99, 1.06]	0.99 ± 0.02 [0.95, 1.03]	2.32, 0.115, 0.006	3.59, **0.039**, 0.028	0.51, 0.728, 0.008
10 m Sprint (s)	1.77 ± 0.03 [1.71, 1.83]	1.72 ± 0.03 [1.66, 1.78]	1.76 ± 0.03 [1.70, 1.81]	1.93, 0.161, 0.010	3.37, **0.047**, 0.046	0.11, 0.977, 0.002
20 m Sprint (s)	3.19 ± 0.04 [3.12, 3.27]	3.29 ± 0.05 [3.20, 3.38]	3.30 ± 0.04 [3.21, 3.38]	30.78, **<0.001**, 0.022	3.65, **0.037**, 0.052	1.82, 0.149, 0.008
Agility (s)	5.00 ± 0.05 [4.89, 5.11]	5.22 ± 0.08 [5.06, 5.38]	5.05 ± 0.06 [4.92, 5.17]	7.89, **0.002**, 0.064	3.17, 0.056, 0.023	1.26, 0.304, 0.019
SJ (cm)	44.95 ± 1.47 [41.96, 47.94]	43.61 ± 1.44 [40.67, 46.55]	43.71 ± 1.38 [40.90, 46.52]	3.06, 0.061, 0.003	3.86, **0.032**, 0.063	0.49, 0.747, 0.002
Standing Long Jump (m)	2.07 ± 0.07 [1.94, 2.21]	2.26 ± 0.10 [2.06, 2.45]	1.91 ± 0.07 [1.76, 2.05]	0.18, 0.832, 0.006	4.37, **0.021**, 0.042	0.93, 0.458, 0.011

Note. EMM = Estimated Marginal Mean; SE = Standard Error; CI = Confidence Interval; R^2^β = Semi-partial R^2^ for fixed effects. F-statistics and *p*-values were obtained from linear mixed models (LMMs). Bold *p*-values indicate statistical significance (*p* < 0.05).

**Table 4:** EMMs for biochemical parameters before and at the end of Ramadan.

Variable	Before Ramadan EMM ± SE	End of Ramadan EMM ± SE	Mean Difference [95% CI]	Time *F*, *p*, *R*^2^*β*	Group *F*, *p*, *R*^2^*β*	Time × Group *F*, *p*, *R*^2^*β*
Albumin (g/dL)	4.65 ± 0.04	4.64 ± 0.04	0.02 [−0.06, 0.09]	0.23, 0.635, 0.007	0.62, 0.546, 0.028	0.42, 0.660, 0.005
Potassium (mmol/L)	4.68 ± 0.07	4.73 ± 0.07	−0.05 [−0.19, 0.10]	0.44, 0.513, 0.005	0.12, 0.888, 0.008	0.26, 0.774, 0.003
Chloride (mmol/L)	103.84 ± 0.26	104.85 ± 0.38	−1.02 [−1.91, −0.12]	5.34, **0.027**, 0.011	1.15, 0.330, 0.007	2.52, 0.096, 0.063
Urea (BUN) (mg/dL)	12.22 ± 0.47	13.96 ± 0.46	−1.74 [−2.74, −0.74]	12.65, **0.001**, 0.069	0.29, 0.753, 0.028	0.94, 0.400, 0.015
BUN/Creatinine Ratio	13.80 ± 0.54	16.37 ± 0.47	−2.57 [−3.78, −1.36]	18.77, **<0.001**, 0.099	1.03, 0.368, 0.009	0.21, 0.808, 0.002

Note. EMM = Estimated Marginal Mean; SE = Standard Error; CI = Confidence Interval; R^2^β = Semi-partial R^2^ for fixed effects. Mean differences were calculated as Before Ramadan—End of Ramadan. F-statistics and *p*-values were obtained from linear mixed models (LMMs). Bold *p*-values indicate statistical significance (*p* < 0.05).

## Data Availability

The original data sets generated and/or analyzed during the current study are not publicly available due to restrictions imposed by the Turkish Ministry of Health, but are available from the corresponding author on reasonable request.

## Abbreviations

The following abbreviations are used in this manuscript:

ACSM	American College of Sports Medicine
AIC	Akaike’s Information Criterion
BeBIS	Nutrition Information System
BUN	Blood Urea Nitrogen
CI	Confidence Interval
EMM	Estimated Marginal Means
HDL	High-Density Lipoprotein
LDL	Low-Density Lipoprotein
LMM	Linear Mixed Models
REML	Restricted Maximum Likelihood
SE	Standard Error
SJ	Squat Jump
TÜBER	Turkish Nutrition Guide
WHR	Waist-to-Hip Ratio

## References

[1] Martínez-Rodríguez A., Miralles-Amorós L., Vicente-Martínez M., Asencio-Mas N., Yañez-Sepúlveda R., Martínez Olcina M. (2022). Ramadan Nutritional Strategy: Professional Soccer Player Case Study. Nutrients.

[2] Zouhal H., Saeidi A., Salhi A., Li H., Essop M.F., Laher I., Rhibi F., Amani-Shalamzari S., Ben Abderrahman A. (2020). Exercise Training and Fasting: Current Insights. Open Access J. Sports Med..

[3] Ghashang S.K., Suwandi A., Buettner M., Hamdan I., Grassl G.A., Gutenbrunner C., Nugraha B. (2024). Alterations in Anthropometric, Inflammatory and Mental Health Parameters during Ramadan Intermittent Fasting in a Group of Healthy People: A Prospective Cohort Study. Front. Nutr..

[4] Abaïdia A.-E., Daab W., Bouzid M.A. (2020). Effects of Ramadan Fasting on Physical Performance: A Systematic Review with Meta-Analysis. Sports Med..

[5] Nobari H., Saedmocheshi S., Murawska-Ciałowicz E., Clemente F.M., Suzuki K., Silva A.F. (2022). Exploring the Effects of Energy Constraints on Performance, Body Composition, Endocrinological/Hematological Biomarkers, and Immune System among Athletes: An Overview of the Fasting State. Nutrients.

[6] Faris M., Jahrami H., Abdelrahim D., Bragazzi N., BaHammam A. (2021). The Effects of Ramadan Intermittent Fasting on Liver Function in Healthy Adults: A Systematic Review, Meta-Analysis, and Meta-Regression. Diabetes Res. Clin. Pract..

[7] Faris M.E., Laher I., Khaled M.B., Zouhal H. (2023). Editorial: The Model of Ramadan Diurnal Intermittent Fasting: Unraveling the Health Implications, Volume II. Front. Nutr..

[8] Brini S., Abderrahman A.B., Clark C.C.T., Zouita S., Hackney A.C., Govindasamy K., Granacher U., Zouhal H. (2021). Sex-Specific Effects of Small-Sided Games in Basketball on Psychometric and Physiological Markers during Ramadan Intermittent Fasting: A Pilot Study. BMC Sports Sci. Med. Rehabil..

[9] Triki R., Zouhal H., Chtourou H., Salhi I., Jebabli N., Saeidi A., Laher I., Hackney A.C., Granacher U., Ben Abderrahman A. (2023). Timing of Resistance Training during Ramadan Fasting and Its Effects on Muscle Strength and Hypertrophy. Int. J. Sports Physiol. Perform..

[10] Ferchichi S., Taktak Y. (2021). Does Ramadan Fasting Affect Diurnal Variation on Swimming Performance and Anaerobic Power?. Eur. J. Phys. Educ. Sport Sci..

[11] Osman F., Haldar S., Henry C.J. (2020). Effects of Time-Restricted Feeding during Ramadan on Dietary Intake, Body Composition and Metabolic Outcomes. Nutrients.

[12] Saddoud A., Khacharem A., H’Mida C., Trabelsi K., Boukhris O., Ammar A., Clark C.C.T., Glenn J.M., Chtourou H., Jarraya M. (2021). Ramadan Observance Is Associated with Impaired Kung-Fu-Specific Decision-Making Skills. Int. J. Environ. Res. Public Health.

[13] Khemila S., Romdhani M., Farjallah M.A., Abid R., Bentouati E., Souissi M.A., Abedelmalek S., Garbarino S., Souissi N. (2023). Effects of Ramadan Fasting on the Diurnal Variations of Physical and Cognitive Performances at Rest and after Exercise in Professional Football Players. Front. Psychol..

[14] Hajji J., Sabah A., Aljaberi M.A., Lin C.-Y., Huang L.-Y. (2023). The Effect of Ramadan Fasting on the Coping Strategies Used by Male Footballers Affiliated with the Tunisian First Professional League. Healthcare.

[15] Farooq A., Chamari K., Sayegh S., El Akoum M., Al-Mohannadi A.S. (2021). Ramadan Daily Intermittent Fasting Reduces Objectively Assessed Habitual Physical Activity among Adults. BMC Public Health.

[16] Brini S., Ardigò L.P., Clemente F.M., Raya-González J., Kurtz J.A., Casazza G.A., Castagna C., Bouassida A., Nobari H. (2023). Increased Game Frequency Period Crossing Ramadan Intermittent Fasting Decreases Fat Mass, Sleep Duration, and Recovery in Male Professional Basketball Players. PeerJ.

[17] Ahmed I., Maughan R.J., Iqbal Z., Ali K., Naji O., Awan S., Tumi A.M., Chamari K. (2022). Competing in the Ramadan Fasted State: For Spirituality, Health and Performance. Br. J. Sports Med..

[18] Kerksick C.M., Wilborn C.D., Roberts M.D., Smith-Ryan A., Kleiner S.M., Jäger R., Collins R., Cooke M., Davis J.N., Galvan E. (2018). ISSN Exercise & Sports Nutrition Review Update: Research & Recommendations. J. Int. Soc. Sports Nutr..

[19] Faris M.E., Laher I., Khaled M.B., Mindikoglu A.L., Zouhal H. (2022). Editorial: The Model of Ramadan Diurnal Intermittent Fasting: Unraveling the Health Implications—Volume I. Front. Nutr..

[20] Correia J.M., Santos I., Pezarat-Correia P., Minderico C., Mendonca G.V. (2020). Effects of Intermittent Fasting on Specific Exercise Performance Outcomes: A Systematic Review Including Meta-Analysis. Nutrients.

[21] Meckel Y., Ismaeel A., Eliakim A. (2008). The Effect of the Ramadan Fast on Physical Performance and Dietary Habits in Adolescent Soccer Players. Eur. J. Appl. Physiol..

[22] Fashi M., Ahmadizad S., Nobari H., Pérez-Gómez J., Oliveira R., Carlos-Vivas J., Ardigò L.P. (2021). Effect of Acute Ramadan Fasting on Muscle Function and Buffering System of Male Athletes. Healthcare.

[23] Chennaoui M., Desgorces F., Drogou C., Boudjemaa B., Tomaszewski A., Depiesse F., Burnat P., Chalabi H., Gomez-Merino D. (2009). Effects of Ramadan Fasting on Physical Performance and Metabolic, Hormonal, and Inflammatory Parameters in Middle-Distance Runners. Appl. Physiol. Nutr. Metab..

[24] Brini S., Castillo D., Raya-González J., Castagna C., Bouassida A., Khalifa R., Chortane S.G., Clemente F.M. (2021). Basketball-Specific Small-Sided Games Training during Ramadan Intermitting Fasting: Do Changes in Body Composition, Sleep Habits, and Perceived Exertion Affect Technical Performance?. Int. J. Environ. Res. Public Health.

[25] Aziz A.R., Che Muhamad A.M., Roslan S.R., Ghulam Mohamed N., Singh R., Chia M.Y.H. (2017). Poorer Intermittent Sprints Performance in Ramadan-Fasted Muslim Footballers despite Controlling for Pre-Exercise Dietary Intake, Sleep and Training Load. Sports.

[26] Zerguini Y., Kirkendall D., Junge A., Dvorak J. (2007). Impact of Ramadan on Physical Performance in Professional Soccer Players. Br. J. Sports Med..

[27] Chtourou H., Hammouda O., Souissi H., Chamari K., Chaouachi A., Souissi N. (2011). The Effect of Ramadan Fasting on Physical Performances, Mood State and Perceived Exertion in Young Footballers. Asian J. Sports Med..

[28] Zerguini Y., Dvorak J., Maughan R.J., Leiper J.B., Bartagi Z., Kirkendall D.T., Al-Riyami M., Junge A. (2008). Influence of Ramadan Fasting on Physiological and Performance Variables in Football Players: Summary of the F-MARC 2006 Ramadan Fasting Study. J. Sports Sci..

[29] Karli U., Guvenc A., Aslan A., Hazir T., Acikada C. (2007). Influence of Ramadan Fasting on Anaerobic Performance and Recovery Following Short Time High Intensity Exercise. J. Sports Sci. Med..

[30] Chaouachi A., Coutts A.J., Chamari K., Wong D.P., Chaouachi M., Chtara M., Roky R., Amri M. (2009). Effect of Ramadan Intermittent Fasting on Aerobic and Anaerobic Performance and Perception of Fatigue in Male Elite Judo Athletes. J. Strength Cond. Res..

[31] Güvenç A. (2011). Effects of Ramadan Fasting on Body Composition, Aerobic Performance and Lactate, Heart Rate and Perceptual Responses in Young Soccer Players. J. Hum. Kinet..

[32] Ali Z., Abizari A.-R. (2018). Ramadan Fasting Alters Food Patterns, Dietary Diversity and Body Weight among Ghanaian Adolescents. Nutr. J..

[33] Trabelsi K., Ammar A., Boukhris O., Glenn J.M., Bott N., Stannard S.R., Engel F.A., Sperlich B., Garbarino S., Chtourou H. (2020). Effects of Ramadan Observance on Dietary Intake and Body Composition of Adolescent Athletes: Systematic Review and Meta-Analysis. Nutrients.

[34] Ministry of Health, General Directorate of Public Health (2022). Türkiye Nutrition Guide (TÜBER).

[35] Thomas D.T., Erdman K.A., Burke L.M. (2016). American College of Sports Medicine Joint Position Statement: Nutrition and Athletic Performance. Med. Sci. Sports Exerc..

[36] Hecht C., Bank N., Cook B., Mistovich R.J. (2024). Nutritional Recommendations for the Young Athlete. J. Pediatr. Orthop. Soc. N. Am..

[37] Mhenni T., Souissi A., Tayech A., Yousfi N., Mejri M.A., Chamari K., Souissi N., Khlifa R., Haddad M. (2021). The Effect of Ramadan Fasting on the Morning-Evening Difference in Team-Handball-Related Short-Term Maximal Physical Performances in Elite Female Team-Handball Players. Chronobiol. Int..

[38] Bouzid M.A., Abaïdia A.-E., Bouchiba M., Ghattassi K., Daab W., Engel F.A., Chtourou H. (2019). Effects of Ramadan Fasting on Recovery Following a Simulated Soccer Match in Professional Soccer Players: A Pilot Study. Front. Physiol..

[39] Guembri M.A., Racil G., Dhouibi M.-A., Coquart J., Souissi N. (2022). Evaluation of Age-Based Sleep Quality and Fitness in Adolescent Female Handball Players. Int. J. Environ. Res. Public Health.

[40] Aloui A., Chtourou H., Hammouda O., Souissi H., Chaouachi A., Chamari K., Souissi N. (2013). Effects of Ramadan on the Diurnal Variations of Physical Performance and Perceived Exertion in Adolescent Soccer Players. Biol. Rhythm Res..

[41] Edwards L.J., Muller K.E., Wolfinger R.D., Qaqish B.F., Schabenberger O. (2008). An R^2^ Statistic for Fixed Effects in the Linear Mixed Model. Stat. Med..

[42] Hammouda O., Chtourou H., Aloui A., Chahed H., Kallel C., Miled A., Chamari K., Chaouachi A., Souissi N. (2013). Concomitant Effects of Ramadan Fasting and Time-of-Day on Apolipoprotein AI, B, Lp-a and Homocysteine Responses during Aerobic Exercise in Tunisian Soccer Players. PLoS ONE.

[43] Hammouda O., Chtourou H., Aloui A., Mejri M., Chahed H., Miled A., Chamari K., Chaouachi A., Souissi N. (2014). Does Ramadan Fasting Affect the Diurnal Variations in Metabolic Responses and Total Antioxidant Capacity during Exercise in Young Soccer Players?. Sport Sci. Health.

[44] Bouhlel E., Denguezli M., Zaouali M., Tabka Z., Shephard R.J. (2008). Ramadan Fasting’s Effect on Plasma Leptin, Adiponectin Concentrations, and Body Composition in Trained Young Men. Int. J. Sport Nutr. Exerc. Metab..

[45] Chaouachi A., Chamari K., Roky R., Wong P., Mbazaa A., Bartagi Z., Amri M. (2008). Lipid Profiles of Judo Athletes during Ramadan. Int. J. Sports Med..

[46] Trabelsi K., Stannard S.R., Ghlissi Z., Maughan R.J., Kallel C., Jamoussi K., Zeghal K.M., Hakim A. (2013). Effect of Fed- versus Fasted-State Resistance Training during Ramadan on Body Composition and Selected Metabolic Parameters in Bodybuilders. J. Int. Soc. Sports Nutr..

[47] Guembri M.A., Racil G., Tounsi M., Aouichaoui C., Russo L., Migliaccio G.M., Trabelsi Y., Souissi N., Padulo J. (2024). Effects of Ramadan Fasting on Sleep and Physical Fitness among Young Female Handball Players. Children.

[48] Trabelsi K., El Abed K., Trepanowski J.F., Stannard S.R., Ghlissi Z., Ghozzi H., Masmoudi L., Jammoussi K., Hakim A. (2011). Effects of Ramadan Fasting on Biochemical and Anthropometric Parameters in Physically Active Men. Asian J. Sports Med..

[49] Trabelsi K., Rebai H., El-Abed K., Stannard S.R., Khannous H., Masmoudi L., Sahnoun Z., Hakim A., Fellman N., Tabka Z. (2011). Effect of Ramadan Fasting on Body Water Status Markers after a Rugby Sevens Match. Asian J. Sports Med..

[50] Al-Jafar R., Wahyuni N.S., Belhaj K., Ersi M.H., Boroghani Z., Alreshidi A., Alkhalaf Z., Elliott P., Tsilidis K.K., Dehghan A. (2023). The Impact of Ramadan Intermittent Fasting on Anthropometric Measurements and Body Composition: Evidence from LORANS Study and a Meta-Analysis. Front. Nutr..

[51] Attarzadeh Hosseini S.R., Motahari Rad M., Hejazi K. (2014). The Effects of Ramadan Fasting and Physical Activity on Body Composition and Hematological Biochemical Parameters. J. Fasting Health.

[52] Zarrouk N., Hammouda O., Latiri I., Adala H., Bouhlel E., Rebai H., Dogui M. (2016). Ramadan Fasting Does Not Adversely Affect Neuromuscular Performances and Reaction Times in Trained Karate Athletes. J. Int. Soc. Sports Nutr..

[53] Rebaï H., Chtourou H., Zarrouk N., Harzallah A., Kanoun I., Dogui M., Souissi N., Tabka Z. (2014). Reducing Resistance Training Volume during Ramadan Improves Muscle Strength and Power in Football Players. Int. J. Sports Med..

